# SNARE proteins rescue impaired autophagic flux in Down syndrome

**DOI:** 10.1371/journal.pone.0223254

**Published:** 2019-11-12

**Authors:** Stefanos Aivazidis, Abhilasha Jain, Abhishek K. Rauniyar, Colin C. Anderson, John O. Marentette, David J. Orlicky, Kristofer S. Fritz, Peter S. Harris, David Siegel, Kenneth N. Maclean, James R. Roede

**Affiliations:** 1 Department of Pharmaceutical Sciences, Skaggs School of Pharmacy and Pharmaceutical Sciences, University of Colorado, Aurora, CO, United States of America; 2 Department of Pathology, University of Colorado School of Medicine, Aurora, CO, United States of America; 3 Department of Pediatrics, University of Colorado School of Medicine, Aurora, CO, United States of America; 4 The Linda Crnic Institute for Down Syndrome, University of Colorado, Aurora, CO, United States of America; University of Nebraska-Lincoln, UNITED STATES

## Abstract

Down syndrome (DS) is a chromosomal disorder caused by trisomy of chromosome 21 (Ts21). Unbalanced karyotypes can lead to dysfunction of the proteostasis network (PN) and disrupted proteostasis is mechanistically associated with multiple DS comorbidities. Autophagy is a critical component of the PN that has not previously been investigated in DS. Based on our previous observations of PN disruption in DS, we investigated possible dysfunction of the autophagic machinery in human DS fibroblasts and other DS cell models. Following induction of autophagy by serum starvation, DS fibroblasts displayed impaired autophagic flux indicated by autophagolysosome accumulation and elevated p62, NBR1, and LC3-II abundance, compared to age- and sex-matched, euploid (CTL) fibroblasts. While lysosomal physiology was unaffected in both groups after serum starvation, we observed decreased basal abundance of the Soluble N-ethylmaleimide-sensitive-factor Attachment protein Receptor (SNARE) family members syntaxin 17 (STX17) and Vesicle Associated Membrane Protein 8 (VAMP8) indicating that decreased autophagic flux in DS is due at least in part to a possible impairment of autophagosome-lysosome fusion. This conclusion was further supported by the observation that over-expression of either STX17 or VAMP8 in DS fibroblasts restored autophagic degradation and reversed p62 accumulation. Collectively, our results indicate that impaired autophagic clearance is a characteristic of DS cells that can be reversed by enhancement of SNARE protein expression and provides further evidence that PN disruption represents a candidate mechanism for multiple aspects of pathogenesis in DS and a possible future target for therapeutic intervention.

## Introduction

**Fig 1:**
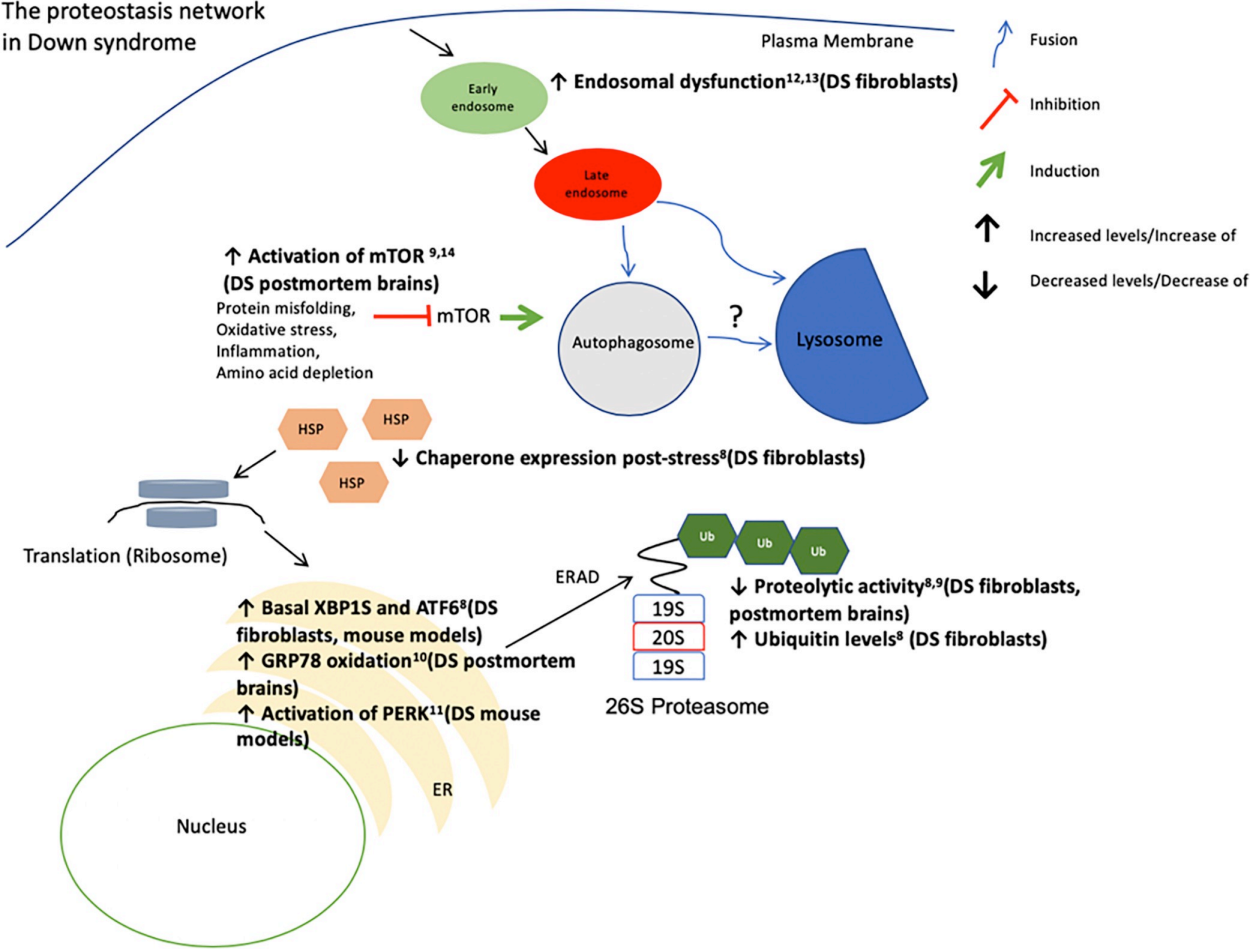
The proteostasis network in Down syndrome. The pathways responsible for preserving proteostasis constitute the proteostasis network (PN). The PN includes the ribosome for proper protein translation, molecular chaperones (HSP) for correct protein folding and protein degradation machinery (26S proteasome for individual peptide degradation and ER-associated degradation (ERAD), autophagy for bulk protein degradation). Its major processes involve successful synthesis, folding, repair/disaggregation and degradation of proteins. In addition, there are several secondary PN modulators, like the unfolded protein response (UPR) caused by endoplasmic reticulum (ER) stress that constitute the stress responsive arm of the PN and provide its plasticity. For extensive reviews of the PN see the following references [15, 81–85].

Down syndrome (DS) is an aneuploidic condition originating from the presence of a third copy of chromosome 21 (Ts21)[1] and is currently the only known trisomy that does not result in early life lethality[2]. DS is characterized by a variable phenotype with multiple comorbidities, including early onset Alzheimer’s disease (EOAD)[1], cognitive disabilities[3], cardiac defects[4], diabetes[5], and immunological dysfunction[6, 7]. Previous research from our laboratory and others revealed the presence of endoplasmic reticulum stress and a dysfunctional proteostasis network (PN) in human DS cells and animal models[8–14] (Fig 1). The observation of multiple independent markers of impaired proteostasis is critical as the PN serves a crucial regulatory role in the cellular proteome by acting to preserve the integrity of protein biogenesis, folding and degradation[15]. Importantly, PN dysfunction has the potential to serve as a pathogenic mechanism for many of the comorbidities that typically occur in DS[16–23].

Since the exact nature of PN dysfunction in DS has yet to be fully elucidated, in the present study we proposed to investigate the effect of DS upon autophagy, which plays a crucial role in the integrity and efficacy of cellular proteostasis. Macroautophagy (hereafter referred to as autophagy) is a major component of the PN and is responsible for bulk protein degradation and elimination of dysfunctional organelles[24]. This process can be activated by multiple stress signals including amino acid starvation, serum starvation, protein aggregation, and oxidative stress and participates in multiple processes at the molecular and organismal level[25–34].

The present study aims to investigate the hypothesis that triplication of chromosome 21 results in significant impairment of autophagic flux in human DS cells compared to euploid control cells. Our data indicates that induction of autophagy via serum starvation results in impaired autophagic flux in human DS cells. These results implicate that impaired autophagic clearance is a characteristic of DS cells that can be reversed by enhancement of SNARE protein expression, while providing further evidence that PN disruption represents a candidate mechanism for multiple aspects of pathogenesis in DS and a possible future therapeutic target.

## Materials and methods

### Reagents and antibodies

The following antibodies were purchased from Abcam: p62 (ab56416), LAMP2A (ab18528), SNAP29 (ab138500), VAMP8 (ab75021). The STX17 antibody was purchased from Proteintech (17815-1-AP). The antibody against LC3B (for western blots and immunofluorescence (IF) with p62) was purchased from Novus Biologics (NB100-2220). The secondary goat anti-rabbit IgG antibody was purchased from R&D systems (HAF008). The Alexa Fluor^®^ 488 secondary goat anti-mouse IgG antibody was purchased from Invitrogen (A11001). The following were purchased from Sigma-Aldrich: Chloroquine diphosphate salt (C6628), and anti-β-actin (A5441). The following antibody was purchased from Cell Signaling: anti-NBR1 (#9891). The following were purchased from Jackson ImmunoResearch: Rhodamine Red^TM^ goat anti-mouse IgG secondary antibody for TRITC (115-295-146), Alexa Fluor^®^ 488 goat anti-rabbit IgG secondary antibody for FITC (111-545-144), HRP goat anti-mouse secondary antibody (115-036-003). The Lysotraker Red DND-99 reagent was purchased from Invitrogen (L752). Cathepsin B activity fluorometric assay kit was purchased from Biovision (#K-140).

### Cell culture

Four pairs of age- and sex-matched euploid and DS fibroblast cell lines were obtained from the Coriell Institute for Medical Research (Table 1). Human, dermal fibroblasts (passage 6 to passage 11) were cultured in Minimum Essential Medium Eagle including L-glutamine and Earle’s salts (Corning, 10-010-CV), with 10% or 15% fetal bovine serum (FBS) (Gibco-A31160601) and 1% non-essential amino acids (Gibco-11140050). For the serum starvation experiments, the cells were treated with Minimum Essential Medium Eagle including L-glutamine and Earle’s salts (Corning, 10-010-CV) for eight hours, without addition of FBS. The concentration of Chloroquine (CQ) for the serum starvation and CQ co-treatment was 30μm. Two induced pluripotent stem cell (iPSC) lines were generated by Dr. David Russel’s laboratory (University of Washington) and were provided by Dr. Christopher Link (University of Colorado, Boulder) in communication with Gretchen Stein[35]. Both iPSC lines originated from the AG06872 fibroblast cell line through reprogramming[36]. These cells lines are isogenic since the trisomic iPSC cell line spontaneously lost the extra 21^st^ chromosome and became euploid during reprogramming and clone selection. iPSCs were cultured on matrigel-coated plates (Corning, 354277) in mTESR^TM^1 (Stem Cell Technologies-#85851) including 5x Supplement (Stem Cell Technologies, 85852). Neural progenitor cells (NPC) were derived by transformation of iPSC cell lines to embryoid bodies, followed by neural induction. Briefly, iPSCs were plated on 60mm ultra-low attachment dishes (Corning, CLS2361) in STEMdiff^TM^ neural induction medium (Stem Cell Technologies, 05839). After formation of embryoid bodies, neuronal rosettes were selected and plated in matrigel-coated 6-well plates in neural induction medium. After 5 days, neural induction medium was replaced with STEMdiff^TM^ neural progenitor basal medium (Stem Cell Technologies, 05834) and neuronal rosettes were left to expand to NPCs.

**Table 1:** Euploid and DS fibroblast cell lines used.

Study ID	Coriell ID	Disease Status	Sex	Age (year)
**Fibroblast**	** **	** **	** **	** **
CTL1	AG04392	CTL	F	Fetal
DS1	AG06872	DS	F	1
CTL2	AG0848	CTL	M	1
DS2	AG06922	DS	M	2
CTL3	AG07095	CTL	M	2
DS3	AG05397	DS	M	1
CTL4	AG04433	CTL	M	Fetal
DS4	AG07438	DS	M	0.75
**iPSC**				
C3	AG06872*	CTL	F	1
C2	AG06872	DS	F	1

* Note: During reprograming this clone lost the third copy of chromosome 21; therefore, it is isogenic to the C2 line.

### Western blotting

For Western blotting, 20–40 μg of each cell homogenate was separated via SDS-PAGE utilizing a 15% polyacrylamide gel. Proteins were transferred to a nitrocellulose membrane using a Trans-Blot Turbo transfer apparatus (Bio-Rad). Membranes were blocked with 5% nonfat dried milk in TBS-0.1% Tween (TBS-T) for 20 minutes. Primary antibodies were diluted in TBS-T containing 10% Super Block T20 (Thermo Scientific, 37536) at appropriate dilutions (1:500–1:1000) and allowed to bind to membranes overnight at 4°C. Blots were washed (3x) for 10 min in TBS-T, the blot was then incubated for 1h at room temperature with a horseradish peroxidase conjugated secondary antibody at 1:5000 or an Alexa Fluor 488^®^ secondary antibody diluted in TBS-T containing 10% Super Block T20. Clarity Western ECL Substrate (Bio-Rad, 1705060) was used to detect the HRP of the secondary antibody. ChemiDoc MP imaging system and Image Lab software (Bio-Rad) were used to image and quantify blots. These experiments were conducted independently at least twice by utilizing 2–3 technical replicates in each experiment, and the images presented are representative samples.

### Immunofluorescence

Cells were plated in a 12-well plate containing glass coverslips (1 coverslip per well) at cell density of 40,000 per well and left to adhere and grow overnight. After treatment, cells were fixed using 3.7% (v/v) paraformaldehyde in PBS and permeabilized using 0.1% (v/v) Triton-X 100 in PBS for 12 minutes. Coverslips were blocked at room temperature using a 1:1 mixture of TBS-T and culture medium (Eagle’s minimal essential medium (EMEM) 15% FBS, 1% Non -essential Amino Acids (NEAA)) for 30 minutes. Then, samples were incubated with a primary antibody overnight at 4°C and washed (3x) using TBS-T. Coverslips were then incubated with TRITC-labeled secondary antibody and/or FITC-labeled secondary antibody and DAPI (1 μg/ml) at room temperature. Next, coverslips were washed (3x) in TBS-T and then mounted on slides using VECTASHIELD anti-fade mounting medium (Vector laboratories, H-1000) and SuperMount (BioGenex, NC9742697) and allowed to dry. Cells were imaged using a Nikon TE2000 microscope with a Nikon C1 confocal imaging system. Each coverslip had five to ten different fields imaged, and each experiment was conducted in at least two independent trials for a total of 10–20 images per staining combination, treatment, and genotype. Blue color was detected at 408 nm. Green color was detected at 488 nm. Red color was detected at 567 nm. Analyses of the confocal images were performed as described previously by Orlicky et al[37]. Briefly, images were converted to TIFF (signal gain was similar for all groups and treatment combinations) and the fluorescence signal of the proteins of interest was quantified in these images and normalized against DAPI by using the 3I Slidebook software program (Intelligent Imaging Innovations, 3I, Denver, Colorado).

### Transmission electron microscopy (TEM)

Cells were plated in a 6-well plate containing glass coverslips (1 coverslip per well) at cell density of 40,000 cells per well and left to grow and adhere overnight. After treatment, the following TEM protocol was followed: Cells were fixed with 2.5% glutaraldehyde for 3 hours, followed by incubation with 1% osmium tetroxide in sodium cacodylate trihydrate buffer (0.05M) phosphate buffer for 1 hour and 1% aqueous uranyl acetate for 30 minutes. Cells were then dehydrated with ethanol and/or propylene oxide at different concentrations and time cycles for a total of 5 hours. For the infiltration, Embed-812 resin mix and/or propylene oxide was used for over 3 days at different concentrations and time cycles. Resin polymerization followed the infiltration step, by 2, 4, 6-Tris (dimethylaminomethyl) phenol (DMP30) addition at the third day for a total of 6 hours. BEEM® capsules for microtomy were inverted on coverslips incubating with Embed-812 and DMP30 and placed in an oven (60°C) for 3 days. Blocks were separated from coverslips using liquid nitrogen to cool, and then heating on a hot plate. Sixty nanometer thin sections were cut on a Leica UC6 ultramicrotome. Images were captured on an FEI T12 Spirit BT (Tecnai) at 100kV. Each sample was imaged ten times (ten different fields) per treatment and genotype. Characterization of autophagolysosome was based on two criteria: a) presence of a double membrane and b) presence of electron-dense regions. Images were randomized, de-identified and then scored by at least two independent assessors blinded to genotype and treatment during autophagolysosome characterization.

### Plasmids and lipofectamine transfection

The plasmids used for the transfection were obtained from Addgene: pt-FLAG (#31385), FLAG-STX17 (#45911), pcDNA3-EGFP (#13031), pEGFP-VAMP8 (#42311). CTL and DS fibroblasts were transfected with Lipofectamine3000 transfection reagent (L3000015) obtained from ThermoFisher Scientific. CTL (AG004392) and DS1 (AG006872) fibroblasts were plated on glass coverslips in 12-well plates with 1 ml of medium per well and left to reach 70% confluency. For each transfection, 2.5 μl of Lipofectamine3000, 1.5 μg of plasmid DNA and 1.5μl of P3000 reagent were diluted in 120 μl of OPTI-MEM reduced serum medium obtained from ThermoFisher Scientific (31985062) and incubated for 15 minutes at room temperature before addition to each well. The medium was replaced with fresh medium the next day and then the cells were used in IF experiments (NT = not treated or SS = 8h serum starvation).

### Lysotracker fluorescence intensity measurement

Cells were plated in a 96-well plate at cell density of 10,000/well and left to adhere and grow overnight. Lysotracker Red was added 45 minutes before the end of the serum starvation treatment (8 hours) at a final concentration of 75 nM. Red fluorescence (Ex/Em: 577nm/590nm) was measured by using a fluorescent plate reader (Molecular Devices). Lysotracker fluorescence intensity was normalized against protein abundance and is reported as a % of Lysotracker fluorescence intensity of the CTL sample after serum starvation.

### Cathepsin B activity assay

Cathepsin B activity assay was performed according to the manufacturer’s protocol. Briefly, cells were plated in 10 cm plates at cell density of 300,000/plate and left to adhere and recover until the plate achieved 80–90% confluence. Cells were treated as per the experimental conditions and then lysed and protein concentration of the samples was measured by BCA assay. 50 μg of cell lysate protein was added to a black, flat-bottom 96 plate (50 μg per well). Reaction buffer and substrate were added in each well and the sample mix was incubated for one hour at room temperature. Negative controls containing a cathepsin-B inhibitor were also used. Fluorescence (Ex/Em: 400nm/505nm) was measured by using a fluorescent plate reader (Molecular Devices). Cathepsin B activity was normalized against protein abundance and is reported as % of Cathepsin B activity of the CTL sample after serum starvation.

### Statistics

Data were analyzed and graphs were plotted using GraphPad Prism 6 software. Data is represented as the mean ± standard error of the mean (SEM). All experiments were repeated independently at least twice, including two to ten technical replicates. Statistical significance in experiments including only CTL1 (AG004392) and DS1 (AG006872) cell lines was determined using unpaired t-test with equal SD. Statistical significance in experiments including all 4 CTL and all 4 DS pairs was determined using paired t-test (pairing based on age and sex). A P-value of <0.05 was deemed to be significant (* P<0.05; ** P<0.01; *** P<0.001; **** P<0.0001).

## Results

### DS fibroblasts exhibit reduced autophagic flux following serum starvation

**Fig 2:**
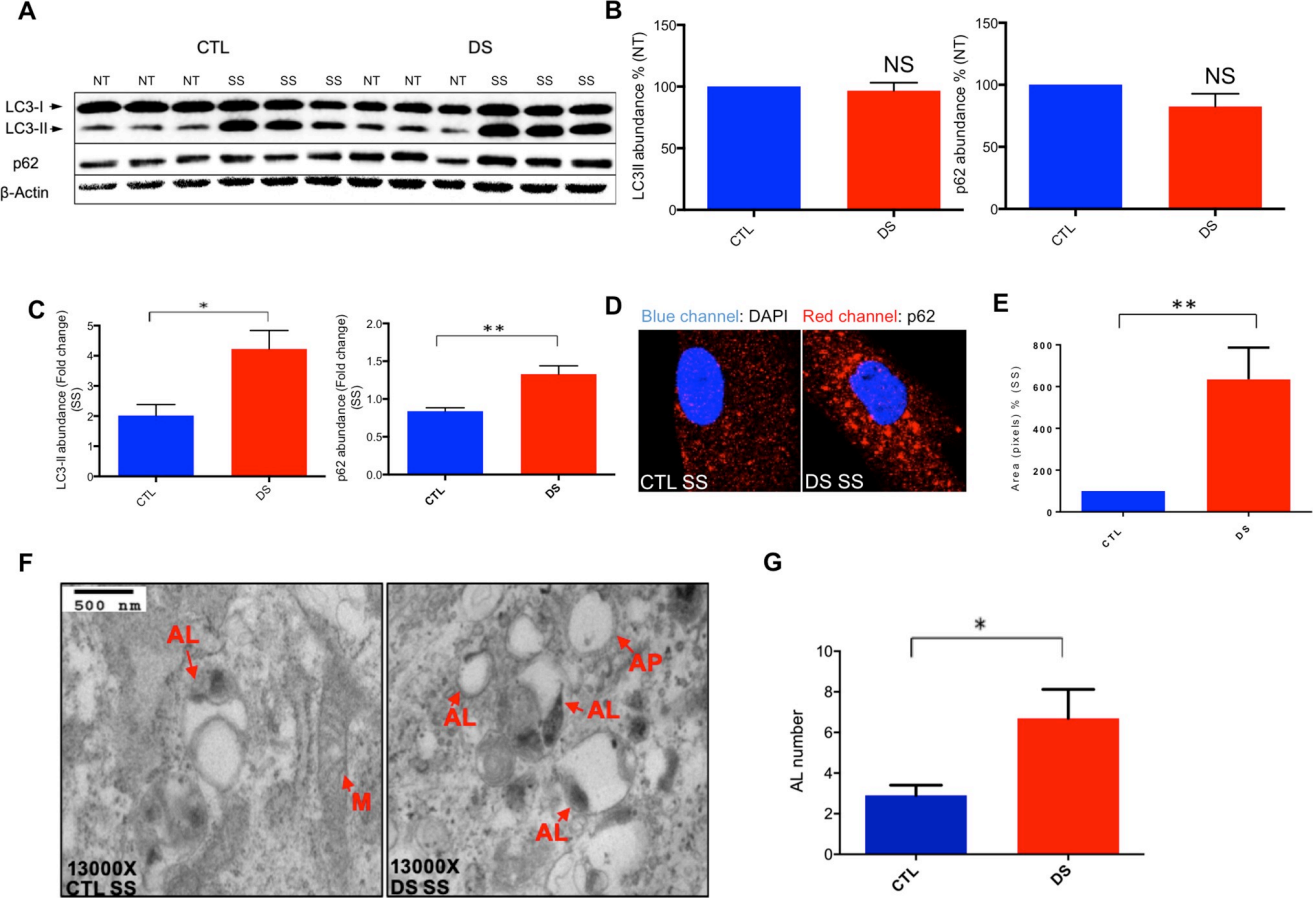
DS fibroblasts exhibit increased autophagolysosome accumulation and elevated abundance of p62 and LC3II after serum starvation. (A) Representative blot of LC3-I, LC3-II, p62 and β-actin of a CTL (AG004392) and DS fibroblast (AG006872) cell line (three technical replicates per treatment and genotype are presented for each cell line). (B) Quantification of abundance levels of LC3-II and p62 at basal levels (% based on abundance of CTL NT) (C) Quantification of fold change in abundance of LC3II and p62 after serum starvation. (D) Immunofluorescence for p62 (Red) and DAPI (blue) following serum starvation of CTL and DS fibroblasts. (E) Quantification of p62 fluorescence intensity (% based on abundance of CTL serum starvation). (F) TEM figures of CTL and DS fibroblasts following serum starvation. (G) Quantitation of autophagolysosome number in the TEM figures. AL, autophagolysosome; AP, autophagosome; M, mitochondria; SS, serum starvation (8h).

A key stage in the autophagic process is the formation of the autophagosome, a double-membrane vesicle that engulfs cytoplasmic material and fuses with the lysosome where the engulfed cargo is degraded[38]. LC3-II, a lipidated form of the microtubule-associated protein LC3-I, is a major component of the autophagosome membrane and serves as a marker of successful autophagosome formation[39]. Further, the engulfed autophagic cargo also includes proteins of the autophagy receptor family, with p62 and NBR1 serving as prominent members[40]. These receptor proteins bind ubiquitinated macromolecules and form the core of the autophagosomal cargo destined for degradation[41]. To investigate possible dysfunction of the autophagic process in human-derived DS cells, we utilized serum starvation to induce autophagy[31, 42]. We employed Western blot analyses to measure serum starvation-mediated alterations in a human-derived, age- and sex-matched DS (AG06872) and euploid CTL (AG04392) fibroblast cell lines. Blots for LC3-II and p62 showed no significant difference in basal LC3-II or p62 abundance (Fig 2A and 2B). These data indicate that DS fibroblasts do not differ in expression of markers of basal autophagy compared to disomic CTL. However, the scale of serum starvation-mediated increase of LC3-II and p62 following serum starvation induction of autophagy was significantly elevated in DS fibroblasts relative to their euploid matched controls indicating a possible impairment in autophagic flux in DS cells (Fig 2C).

These serum starvation-mediated results were further confirmed when we chose to include a larger number of fibroblast pairs (Table 1) to extend our analyses (four age- and sex-matched pairs, including AG004392, AG006872). Western blots showed no significant difference in basal LC3-II or p62 abundance between the two groups; however, serum starvation resulted in increased LC3-II and p62 abundance in DS compared to CTL cells (S1 Fig). Consistent with our protein abundance results, immunofluorescence analysis of p62 expression revealed significantly increased levels of this autophagy receptor in DS fibroblasts after serum starvation compared to the euploid CTL group (Fig 2D and 2E). At this point it has to be noted that LC3-II levels were significantly different between groups only by paired t-test (no significance was observed with unpaired t-test analysis when all pairs were incorporated). Nevertheless, it has to be considered that our fibroblast pairs are age- and sex-matched, a fact that justifies our option to use paired t-test analysis. Also, LC3-II abundance in models of impaired autophagic flux can serve as a proxy for both newly created and undegraded autophagosomes. This is why measurement of LC3-II abundance has to be accompanied by p62 examination. In our data, p62 levels in DS fibroblasts were significantly increased compared to the CTL group following paired or unpaired t-test. Therefore, p62 accumulation in DS fibroblasts is an appropriate marker of reduced autophagic flux.

In order further investigate these findings, we analyzed p62 accumulation in a DS iPSC line (C2) and a DS NPC line (C2-derived) (S2 Fig). In both cases, we observed a significant increase in p62 compared to controls. Subsequent analyses also determined p62 protein levels in CTL and DS fibroblasts at multiple time points following serum starvation. This experiment revealed that DS fibroblasts accumulate p62 by 8h post serum starvation, and that this increase persists even after 24h (S3 Fig).

To confirm that the observed p62 accumulation is due to diminished flux of autophagy and is not just an effect of p62 metabolism, we evaluated the relative abundance levels of an additional autophagy receptor, NBR1. Interestingly, immunofluorescence for NBR1 demonstrated that this protein follows a similar pattern to p62 with a significant increase in abundance in the DS fibroblasts after serum starvation compared to diploid CTL (S4 Fig). This result indicates that elevated accumulation is not just specific to p62 and that other cellular autophagy receptors exhibit the same pattern. Collectively, these results are consistent with our hypothesis that poor autophagic flux and subsequent PN dysfunction are features of Ts21 cells and are present at multiple stages of differentiation.

### DS fibroblasts exhibit increased levels of autophagolysosome accumulation consistent with impaired autophagic flux following serum starvation

Impairment of the ability of the cell to clear autophagic cargo results in autophagosome and/or autophagolysosome accumulation. This phenomenon has been implicated in the etiology of multiple conditions including AD, diabetes, Huntington’s disease, and Parkinson’s disease (PD)[20, 43–46]. To further examine autophagic degradation efficiency in DS, we conducted transmission electron microscopy (TEM) studies in DS and euploid CTL fibroblasts; measuring the relative number of autophagolysosome in serum starvation treated cells. After a blinded assessment of the TEM images, these experiments revealed a marked increase of autophagolysosomes in DS cells compared to euploid CTL (Fig 2F and 2G). Given that increased presence of autophagolysosomes is a well-defined index of impaired autophagic flux[47], these results are consistent with our Western blot and immunofluorescence data, adding further evidence of impaired autophagic flux in DS.

### Co-localization of p62 with LC3B and LAMP2A further confirms the presence of undegraded autophagosomes and autophagolysosomes

**Fig 3:**
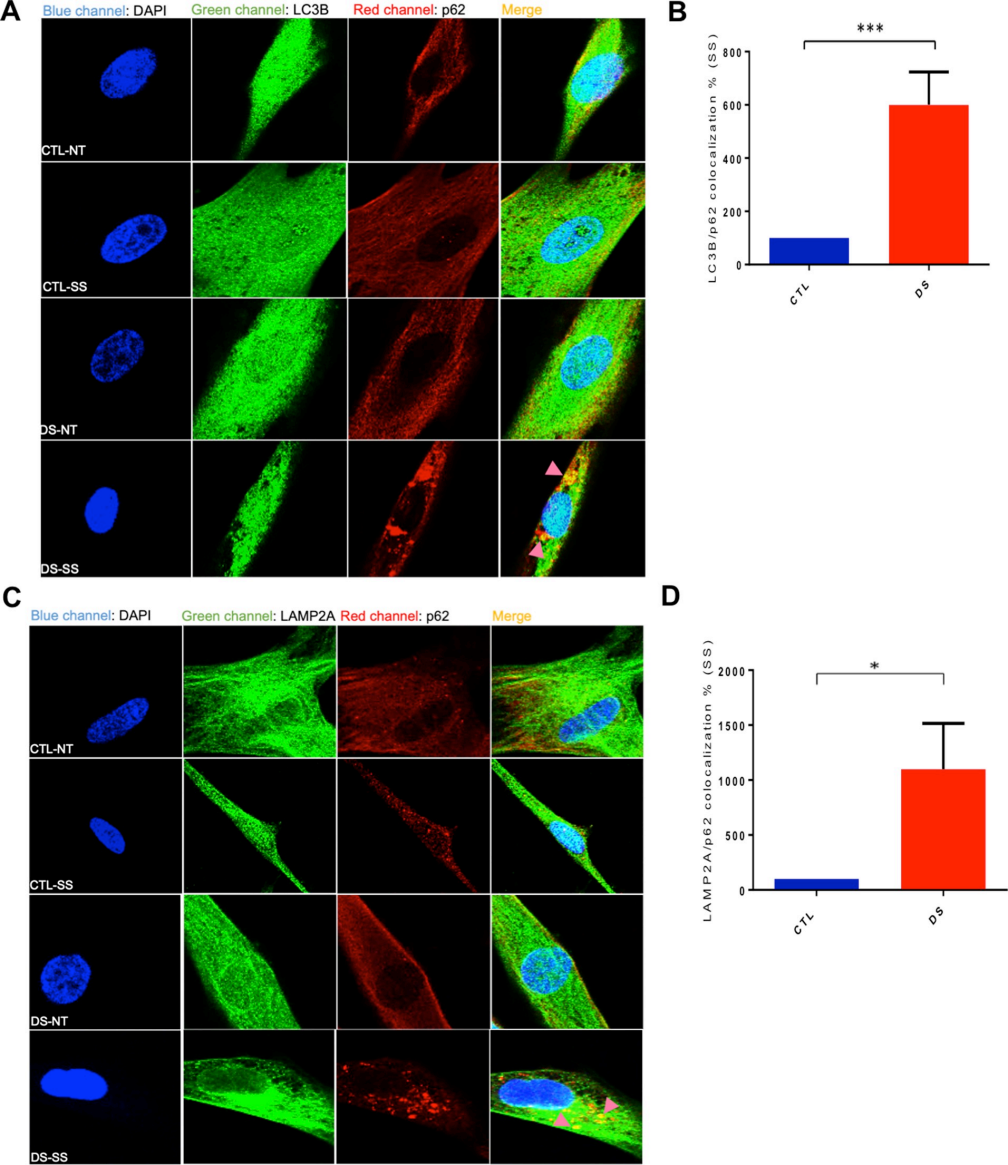
Colocalization of p62 with LC3B and LAMP2A confirms the presence of undegraded autophagosomes and autophagolysosomes. (A) IF for LC3B (Green), p62 (Red) and DAPI (Blue) in a CTL (AG004392) and DS (AG006872) fibroblast cell line at basal levels or after serum starvation. (B) Quantification of LC3B/p62 co-localization (yellow) fluorescence intensity (area-pixels) after serum starvation (% based on abundance of CTL serum starvation). Pink arrow = p62/LC3B co-localized puncta. NT, Not treated-basal levels; SS, serum starvation (8h). (C) IF for LAMP2A (Green), p62 (Red) and DAPI (Blue) in a CTL and DS fibroblast cell line at basal levels or after serum starvation. (D) Quantification of LAMP2A/p62 co-localization (yellow) fluorescence intensity (area-pixels) after serum starvation (% based on abundance of CTL serum starvation). Pink arrow = p62/LAMP2A co-localized puncta.

LC3B is a ubiquitin-like protein that covalently attaches to phosphatidylethanolamine in the autophagosomal membrane where it functions in autophagosome biogenesis, recruitment of cargo and plays a crucial role in fusion with the lysosome. Because co-localization of LC3B and p62 is indicative of impeded autophagic flux and accumulation of undegraded autophagosomes, we performed an immunofluorescence co-localization study for p62 and LC3B following serum starvation. In agreement with previous experiments, co-localization was only observed in DS cells and was completely absent from CTL disomic cells (Fig 3A and 3B). This result further supports our hypothesis that DS cells exhibit a dysfunctional PN and impaired autophagic clearance.

The lysosome-associated membrane protein 2A (LAMP2A) is ubiquitously expressed in lysosomes, is a well characterized marker of lysosomal localization, and is thought to promote membrane integrity for lysosomal stability[48]. We further assessed autophagolysosome accumulation by investigating co-localization of p62 and LAMP2A. Our experiments showed that serum starvation lead to co-localization of p62 with LAMP2A in a DS fibroblast cell line but not in disomic CTL (Fig 3C and 3D), adding further supporting evidence of autophagolysosome accumulation in DS cells.

### Lysosomal dysfunction is not responsible for impaired autophagic flux in DS fibroblasts after serum starvation

**Fig 4:**
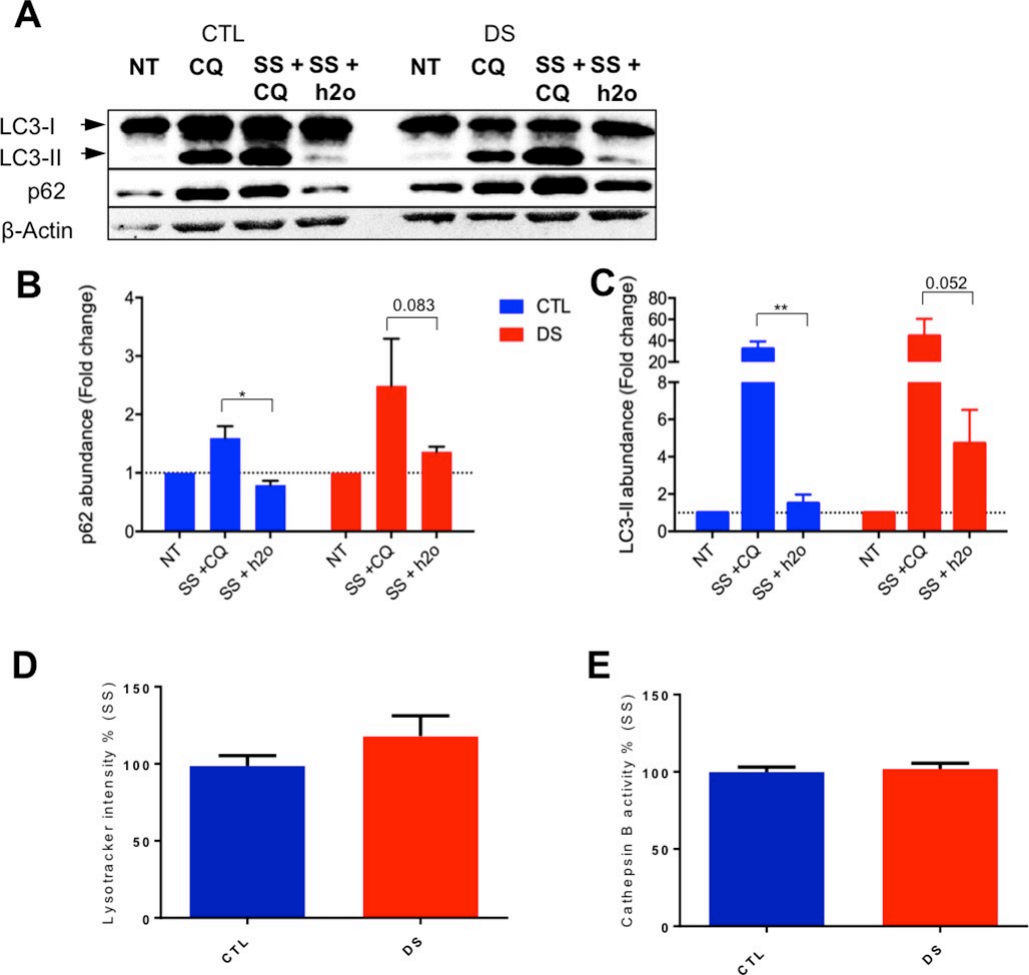
Lysosomal dysfunction is not responsible for p62 accumulation in DS fibroblasts after serum starvation. (A) Representative blot of LC3-I, LC3-II, p62 and β-actin of a CTL (AG004392) and DS (AG006872) fibroblast cell line at basal levels, after CQ treatment (dosage: 30 um), after serum starvation and CQ co-treatment and after serum starvation and H_2_O co-treatment used as control. (B) Quantification of fold change in abundance of p62 of a CTL and DS fibroblast cell line after CQ, serum starvation+CQ and serum starvation+H_2_O. (C) Quantification of fold change in abundance of LC3-II of a CTL and DS fibroblast cell line after CQ, serum starvation+CQ and serum starvation+H_2_O. (D) Lysotracker-Red fluorescence intensity in a CTL and DS fibroblast cell line after serum starvation (% based on fluorescence intensity of CTL serum starvation). (E) Proteolytic activity of lysosomal cathepsin B (% based on activity of CTL serum starvation).

Motivated by our findings, we next evaluated lysosomal endpoints to examine autophagolysosome accumulation and possible lysosomal dysfunction. For these assessments, CTL and DS fibroblasts were co-treated with the lysosomal inhibitor chloroquine (CQ) and serum starvation. CQ is a well-characterized inhibitor of lysosomal proteolytic activity as it alters lysosomal pH and renders lysosomal resident proteases inactive[49]. Therefore, if lysosomal dysfunction is responsible for the observed impairment of autophagic degradation in DS cells, serum starvation co-treatment with CQ or serum starvation alone should display similar levels of p62 and LC3-II in these DS cells. We observed that CQ and serum starvation co-treatment resulted in increased abundance of p62 and LC3-II compared to serum starvation treatment alone (Fig 4A, 4B and 4C) in both groups, indicating similar lysosomal function.

Lysotracker is a lysomotropic dye that emits fluorescence when entering acidic organelles, like lysosomes and late endosomes[50]. To expand our examination of the effect of serum starvation on lysosomal function and abundance and possible pH disturbance in DS and control cells, lysotracker fluorescence intensity was measured in CTL and DS fibroblasts after serum starvation. No significant difference in fluorescence intensity was observed in the CTL or DS fibroblasts, indicating similar lysosomal abundance and lysosomal pH levels between the two groups (Fig 4C).

In order to perform its degradative function, the lysosome contains proteolytic enzymes called cathepsins[51, 52] and these enzymes are functional exclusively in the lysosomal space due to the required acidic pH conditions. To further investigate the possible impact of Ts21 upon lysosomal physiology, we evaluated cathepsin B function as an index of lysosomal degradation capacity since inhibition of this activity could explain dysfunctional clearance in DS fibroblasts. Cathepsin B activity did not differ significantly between CTL and DS cells after serum starvation, indicating that lysosomal proteolysis in DS cells is not fundamentally impaired (Fig 4D). Collectively, our data indicates that lysosomal dysfunction is unlikely to be contributing to the observed accumulation of autophagosomes and autophagolysosomes and impaired autophagic flux in DS fibroblasts.

### DS cell exhibit significantly decreased levels of the SNARE proteins STX17 and VAMP8

**Fig 5:**
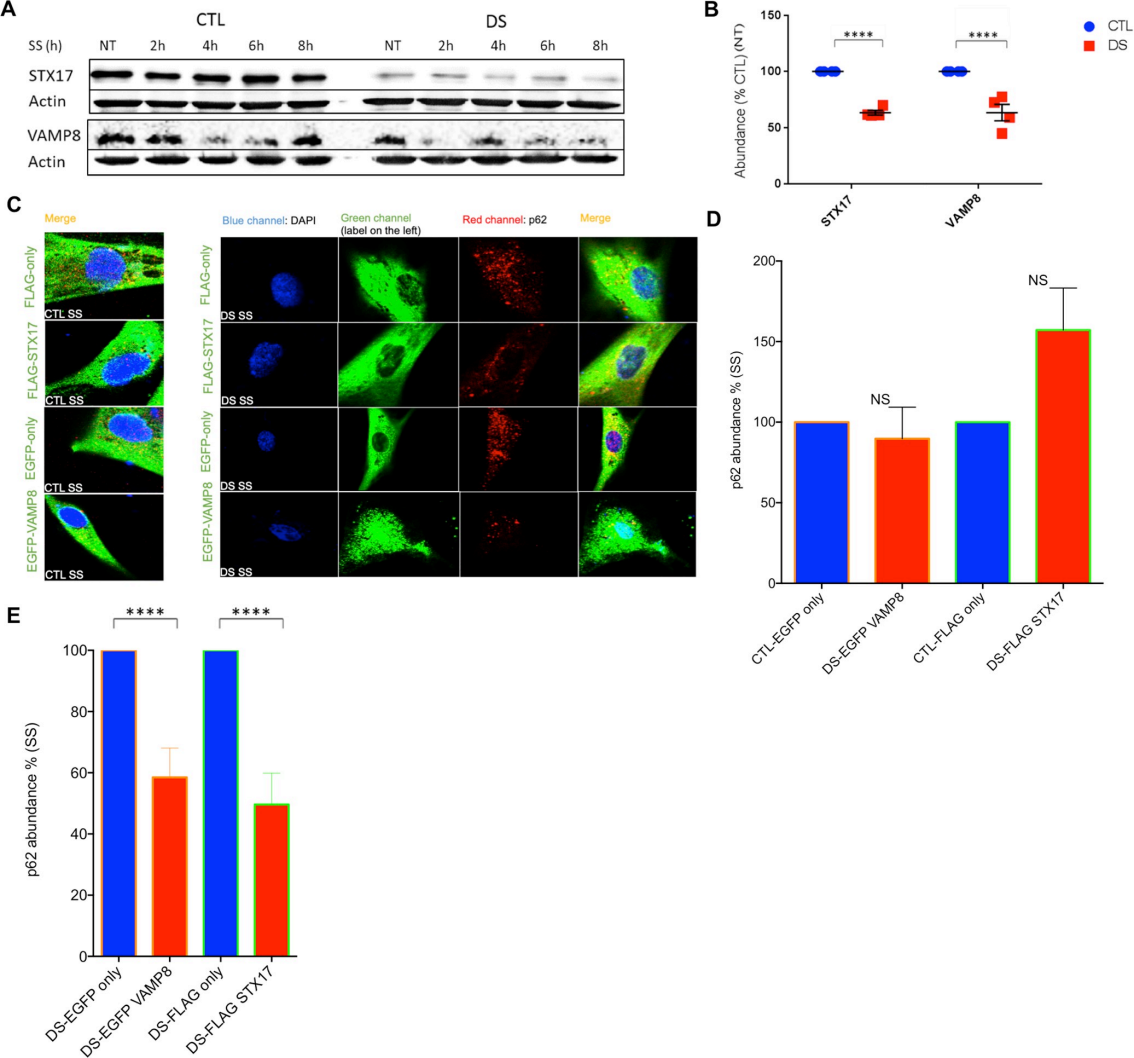
Overexpression of STX17 and VAMP8 restores autophagic flux in DS cells after serum starvation. (A) Representative blot of STX17 and β-actin or VAMP8 and β-actin of CTL and DS fibroblast cell line at basal levels (NT) or after serum starvation (2h-8h). (B) Quantification of STX17 and VAMP8 abundance (%CTL) at basal levels. (C) IF in a CTL (AG004392) and DS (AG006872) fibroblast cell line CTL and DS fibroblast cell line after serum starvation for FLAG-only, FLAG-STX17, EGFP-only, EGFP-VAMP8 (green), p62 (Red) and DAPI (Blue). (D) Quantification of p62 fluorescence intensity (area-pixels) after serum starvation in DS (AG006872) fibroblast cell line transfected with EGFP-VAMP8 vector (% based on p62 abundance of CTL cells transfected with EGFP-only vector). Quantification of p62 fluorescence intensity (area-pixels) after serum starvation and in a DS (AG006872) fibroblast cell line transfected with FLAG-STX17 vector (% based on abundance of CTL cells transfected with FLAG-only EV) (E) Quantification of p62 fluorescence intensity (area-pixels) after serum starvation in DS (AG006872) fibroblasts transfected with FLAG-only or FLAG-STX17 vector (% based on abundance of DS cells transfected with FLAG-only vector). Quantification of p62 fluorescence intensity (area-pixels) after serum starvation in DS (AG006872) fibroblasts transfected with EGFP-only or EGFP-VAMP8 vector (% based on abundance of DS cells transfected with FLAG-only vector).

A crucial group of proteins in autophagy are the members of the SNARE protein family, with greater than 60 members in mammalian cells[53]. These proteins play a crucial role in vesicle fusion with target, membrane-bound compartments[54, 55]. Regarding autophagy, three SNARE proteins, STX17, VAMP8 and SNAP29, are involved in the fusion of the autophagosome with the late endosome/lysosome. Specifically, STX17 binds to the autophagosome membrane only after the closure of the autophagosomal double membrane and completion of autophagosome maturation[56]. STX17 interacts with SNAP-29 and the late endosome/lysosome SNARE protein, VAMP8. This interaction facilitates autophagosome fusion with the late endosome/lysosome. As inhibited function of the STX17-SNAP29-VAMP8 complex can disturb autophagic flux and promote or exacerbate pathogenesis[57], we next examined the abundance of these proteins in DS and control fibroblasts. Although SNAP29 levels were found to be similar between CTL and DS fibroblasts at basal levels (S5 Fig), DS cells displayed significantly reduced (~50–60%) abundance of the autophagosome SNARE protein STX17 and the lysosomal SNARE protein VAMP8 (Fig 5A and 5B). These proteins have a crucial role in autophagosome-lysosome fusion and their decreased abundance offers a plausible explanation for the presence of undegraded ALs in DS cells after serum starvation.

### Overexpression of STX17 and VAMP8 restores autophagic flux in DS cells following serum starvation

In order to further investigate the possibility that decreased abundance of SNARE proteins is implicated in the observed impairment of autophagic clearance in DS cells, FLAG-STX17 or EGFP-VAMP8 were transiently overexpressed in CTL and DS fibroblasts and p62 abundance after serum starvation was measured by immunofluorescence. In this study we observed that over-expression of either STX17 or VAMP8 prevented the previously observed p62 accumulation in DS fibroblasts and restored normal autophagic flux after serum starvation (Fig 5C and 5D). The level of p62 abundance in DS cells over-expressing either STX17 or VAMP8, post-serum starvation level was found to be essentially identical to that observed in control cells transfected with the relevant empty vector (Fig 5D). Similarly, DS fibroblasts overexpressing VAMP8 or STX17 exhibited significantly reduced p62 levels following serum starvation treatment compared to DS cells transfected with the empty vector (Fig 5E). Together, our findings indicate that overexpression of either STX17 or VAMP8 acts to rescue impaired autophagic flux in DS fibroblasts following serum starvation. Furthermore, low SNARE protein abundance in DS fibroblasts is a plausible mechanism for dysfunctional autophagosome fusion with the lysosome and concomitant impaired autophagic flux and p62 accumulation in DS.

## Discussion

The presence of an extra copy of chromosome 21 in the DS genome puts DS individuals at high risk for developing certain comorbidities, such as AD, immune dysfunction, diabetes and leukemia[1, 5–7, 58–60]. Many of the DS comorbidities are associated with a dysfunctional PN[16–23] and aneuploidy has also been associated with proteotoxic stress and PN dysfunction[61–69]. These findings inspired our interest in investigating possible PN dysfunction and the presence of a dysregulated protein quality control system in DS. Recently, our laboratory has reported dysfunctional PN in DS models with increased presence of ER stress, limited chaperone expression after heat stress and increased sensitivity to proteotoxic compounds[8]. Data in this report builds upon our previous research and focuses on further characterizing the possible pathogenic role of PN disruption through the study of the autophagic process in DS cells.

Western blot results showed that DS and euploid control cells possess similar basal levels of LC3-II and p62, indicating that DS cells do not exhibit impaired autophagic flux at basal levels. These data indicated a necessity to interrogate stimulus-induced autophagy to confirm or deny if this key process is dysfunctional in a model that already displays markers of altered proteostasis. Our rationale for the use of SS instead of chemical induction, e.g Torin1 or Rapamycin, was that it is the least physiologically intrusive method and least molecularly promiscuous strategy to induce autophagy. Studies are ongoing in our laboratory to investigate the impact that Ts21 has on autophagy induced by these alternate methods. Serum starvation-mediated increased abundance of LC3-II and autophagy receptors, p62 and NBR1, observed in the DS cell models is consistent with diminished autophagic flux and autophagosome/autophagolysosome accumulation. Additionally, TEM analyses revealed an increased presence of autophagolysosome in DS fibroblasts following serum starvation, and this autophagolysosome buildup is also consistent with our Western blot and immunofluorescence results. Moreover, the observation of impeded autophagic clearance of p62 in several DS cell models at different stages of differentiation, e.g. fibroblast, iPSC and NPC, also suggests that this impairment may occur in a relatively wide range of DS cell types and at multiple stages of development and differentiation. Data from the C2 and C3 iPSC lines further implicate Ts21 as a mechanistic component of the disrupted autophagic flux because these two cell lines are isogenic and differ only in the number of copies of chromosome 21 (2 vs. 3). Together, the significant persistence of autophagosome/autophagolysosome accumulation markers throughout our experiments provides supporting evidence implicating decreased autophagic flux as a cellular phenotype of DS.

For further characterization of the autophagic process in DS cells it was pertinent to evaluate possible autophagosome and/or autophagolysosome buildup as a result of abnormal autophagic flux. Co-localization of p62 with LC3B and LAMP2A appeared only in DS fibroblasts after serum starvation, confirming autophagosome and autophagolysosome accumulation. Furthermore, the observation that serum starvation co-treatment with CQ resulted in an increased abundance of both p62 and LC3-II in DS compared to the serum starvation-only treatment indicates that the lysosome is most likely not responsible for the inhibition of autophagic clearance. Adding further weight to this conclusion are the observations of normal lysosomal pH levels and cathepsin B activity in both CTL and DS fibroblasts following serum starvation. Collectively, our data suggest that, in the absence of lysosomal physiology disruption, limited autophagosome-lysosome fusion might be responsible for autophagic flux defects.

The importance of SNARE proteins in autophagy has been repeatedly reported in the literature[54, 70–72], particularly in regards to their role in autophagosome-lysosome fusion. As mentioned before, STX17 interacts with SNAP29 and VAMP8 to aid in autophagosome fusion to the target membrane of the lysosome[55]. Our research reports decreased abundance of SNARE proteins in DS cells for the first time and this narrative is further strengthened by the observation that impaired autophagic flux in DS cells can be repaired by induced overexpression of either STX17 or VAMP8. The cause of reduced STX17 and VAMP8 basal abundance in DS cells is an enigma that is outside of the scope of this manuscript and will be investigated in future research endeavors. Increasing the abundance of either of these two SNARE proteins reduced p62 levels (~50%) compared to DS fibroblasts transfected with empty vectors in serum starvation conditions. Also, p62 levels did not differ significantly compared to CTL cells overexpressing empty vectors in serum starvation conditions, indicating reconstitution of proper autophagic flux in the DS cells. The decreased basal abundance of trafficking/tethering/fusion mediators can serve a causative factor for limited flux and possibly endosomal dysfunction observed in DS models[12, 13, 73]. Furthermore, research in cells involving mutant versions of STX17 has shown autophagosome accumulation as a primary outcome[56], while previous work in Drosophila models carrying a STX17 mutation[74] also exhibit autophagosome and autophagolysosome increase after starvation in a manner consistent with our observation of autophagosome and autophagolysosome accumulation in DS models after serum starvation.

**Fig 6:**
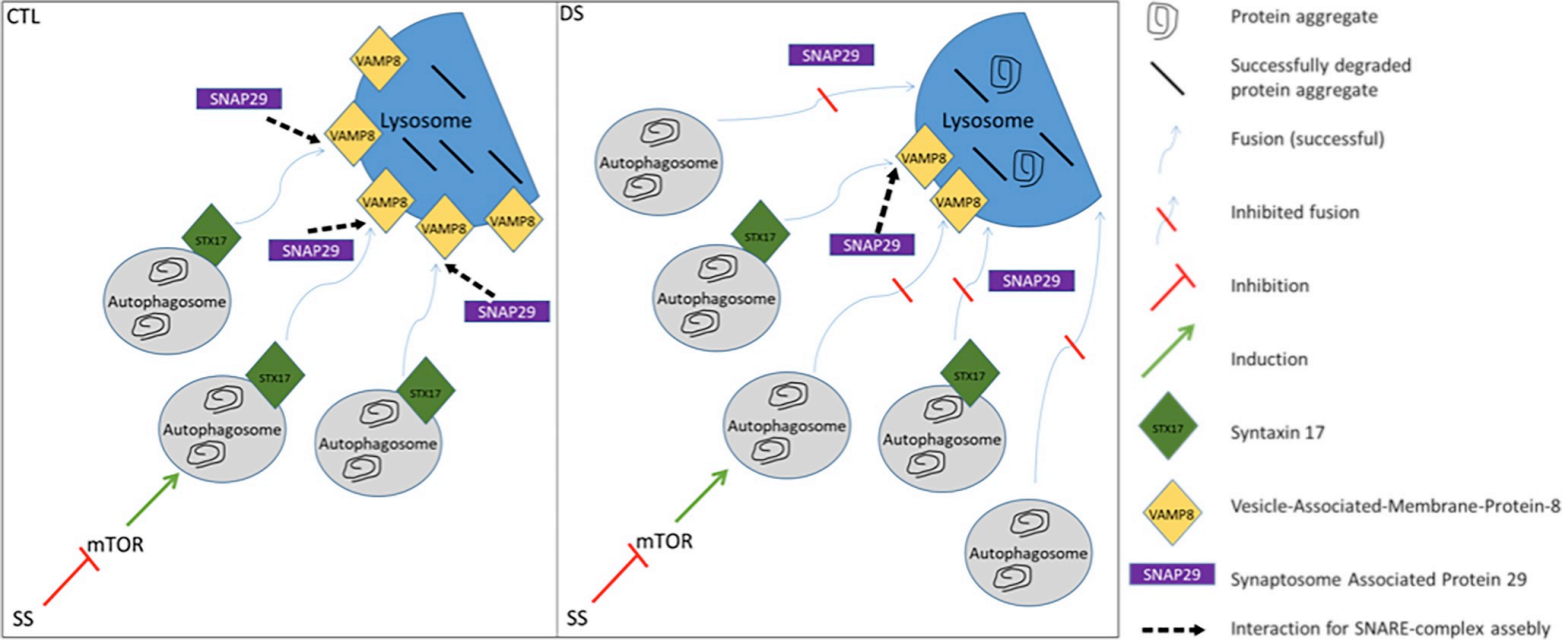
Diminished autophagic flux in DS fibroblasts after serum starvation is characterized by autophagosome fusion defects due to reduced STX17 and VAMP8 levels (SS = serum starvation, 8h).

Informed by the data presented in this report, we propose a model of diminished autophagic flux due to reduced fusion between autophagosome and lysosomes (Fig 6). Low abundance of STX17 and VAMP8 leads to partially impaired fusion, resulting in autophagosome/autophagolysosome accumulation and increased abundance of LC3II, p62 and NBR1. Overall, the changes in autophagic flux in DS are relatively subtle compared to the more profound dysfunction involved in the severe comorbidities of lysosomal storage disorders (LSDs), e.g. parkinsonism in Gaucher disease[75, 76], liver failure with 4 month life expectancy in Wolman’s disease [77]. However, LSDs and DS share similar comorbidities, like neurodegeneration[78] and musculoskeletal disorders[79]. To add to this, a study focusing on the lipid storage disorder Niemann-Pick type C1 reported diminished autophagic flux in the presence of unaffected lysosomal proteolysis[80], which is consistent with the results presented here. It should be noted that the onset of related comorbidities appear earlier in LSDs compared to DS consistent with the relatively mild impairment of autophagic flux in DS cells. Further investigation is needed to define possible molecular signatures of autophagic dysfunction in DS, such as localization and relative kinetics of the various molecular events involved and the effect of aging on PN collapse.

In summary, our results indicate that diminished autophagic flux is a characteristic of DS cells that can be reversed by enhancement of SNARE protein expression. Furthermore, our findings indicate that subsequent PN disruption can serve as a candidate cellular mechanism for multiple aspects of pathogenesis in DS models [8] and has the potential to represent a novel target for therapeutic intervention.

## Supporting information

S1 File This file contains the whole blot images for the Western blots presented in this manuscript. (PDF)

S1 Fig Increased fold change in LC3II and p62 abundance after serum starvation is observed in CTL and DS fibroblasts. (A) Representative blot of LC3-I, LC3-II, p62 and β-actin of CTL1-4 and DS1-4 fibroblast cell lines (three technical replicates per treatment and genotype of one CTL and DS cell line are presented). (B) Quantification of abundance levels of LC3-II and p62 of four cell line pairs of CTL and DS fibroblasts at basal levels (% based on abundance of CTL NT) (C) Quantification of fold change in abundance of LC3II and p62 of four cell line pairs of CTL and DS fibroblasts after serum starvation
(TIF)

S2 Fig Increased fold change in p62 abundance after serum starvation is observed in alternative DS cell models. (A) Representative blots for p62 and b-actin of a CTL and DS iPSC cell line at basal levels (NT) or after serum starvation (three technical replicates per treatment and genotype of one CTL and DS cell line are presented). (B) Quantification of fold change in abundance of p62 after serum starvation. (C) Representative blots for p62 and b-actin of a CTL and DS NPC cell line at basal levels or after serum starvation. (D) Quantification of fold change in abundance of p62.
(TIF)

S3 Fig DS fibroblasts exhibit significantly increased fold change in abundance of p62 after 8h and 12h of serum starvation compared to CTL. Western blots were conducted over a 24h period to investigate the temporal changes in p62 protein levels after serum starvation in a CTL (AG004392-Blue) and DS (AG006872-Red) fibroblast cell line. Quantification of fold change in abundance of p62 at 0h, 4h, 8h, 12h, 16h or 24h of serum starvation. Statistical analysis was performed by paired t-test analysis at each individual time point.
(TIF)

S4 Fig NBR1 abundance is significantly higher in DS fibroblasts after serum starvation. (A) Immunofluorescence for NBR1 (green) and DAPI (blue) in a CTL (AG004392) and DS (AG006872) fibroblast cell line at basal levels or after serum starvation. (B) Quantification of NBR1 (green) fluorescence intensity (area-pixels) after serum starvation (% based on abundance of CTL serum starvation). NT, Not treated-basal levels; SS, Serum starvation (8h).
(TIF)

S5 Fig SNAP29 levels are not significantly different between CTL and DS fibroblasts. (A) Representative blot for SNAP29 and β-actin in four CTL and DS fibroblast cell lines (CTL1-4, DS1-4) at basal levels. (B) Quantification of SNAP29 levels between CTL and DS groups. NT, Not treated-basal levels
(TIF)

## Abbreviations

AD: Alzheimer’s disease
AL: autophagolysosome
AP: autophagosome
CQ: Chloroquine diphosphate
CTL: Euploid control
DS: Down syndrome
EOAD: Early onset Alzheimer’s disease
HD: Huntington’s disease
IF: Immunofluorescence/Immunocytochemistry
iPSC: Induced pluripotent stem cell
Lysosomal: storage disorders, LSDs
mTOR: Mammalian target of rapamycin
NBR1: Neighbor Of BRCA1 gene 1
NPC: Neuroprogenitor cells
NT: Basal levels/not treated
PD: Parkinson’s disease
PN: Proteostasis network
STX17: Syntaxin 17
SNAP29: Synaptosome associated protein 29
SNARE: Soluble N-ethylmaleimide-sensitive-factor attachment protein receptor
TEM: Transmission electron microscopy
Ts21: Trisomy 21
VAMP8: Vesicle associated membrane protein 8
