# Supplementary material for: SNARE proteins rescue impaired autophagic flux in Down syndrome
Source: PLoS One. 2019 Nov 12;14(11):e0223254. doi: 10.1371/journal.pone.0223254 (PMC6850524; doi:10.1371/journal.pone.0223254)

Figure 2A – Full blot images

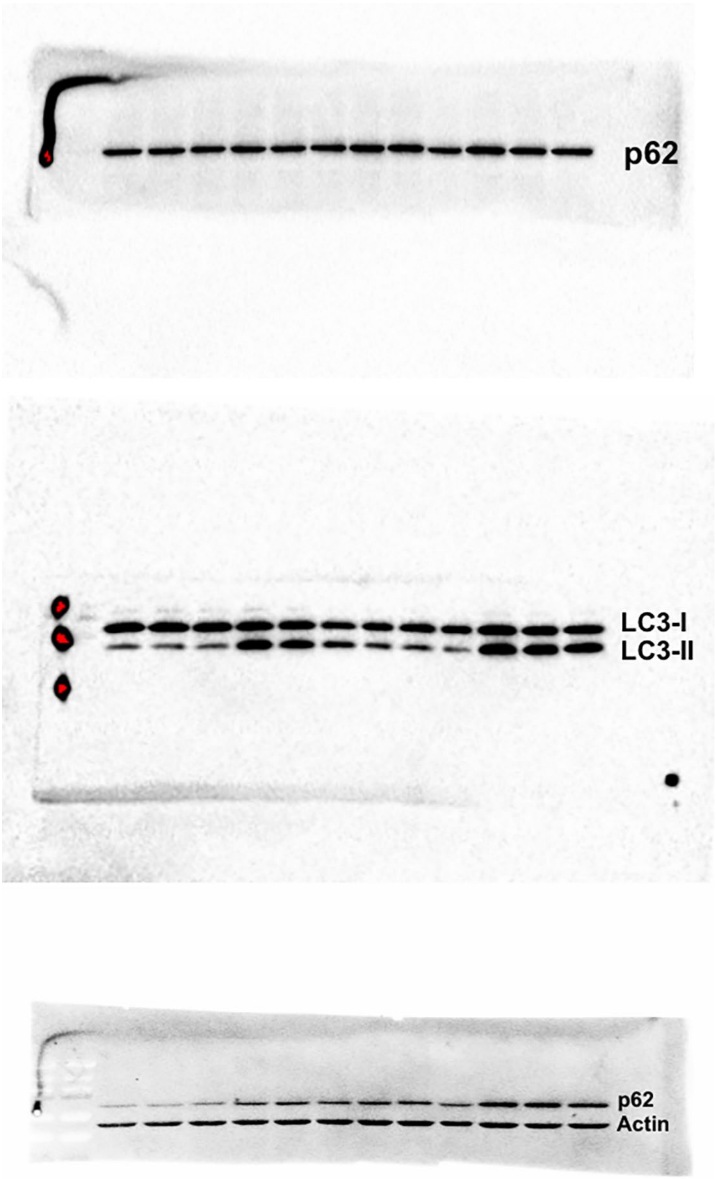

Figure 4A – Full blot images

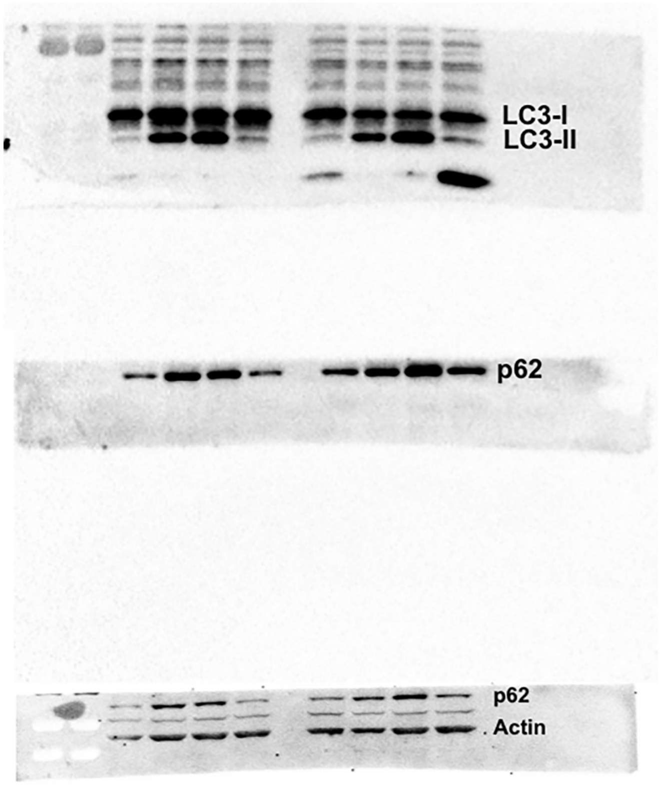

Figure 5A – Full blot images

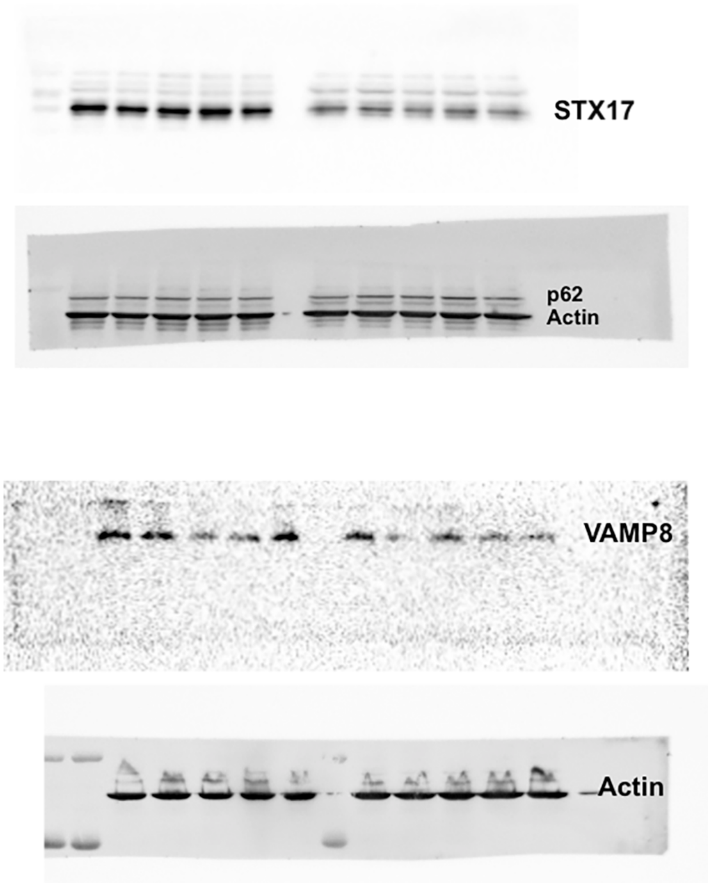

Supplemental Figure 1A – Full blot images

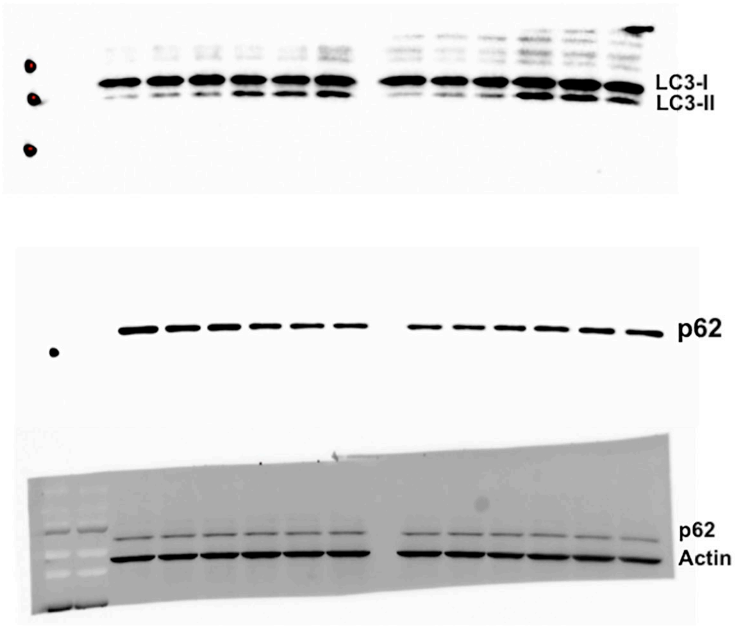

Supplemental Figure 2A&C – Full blot images

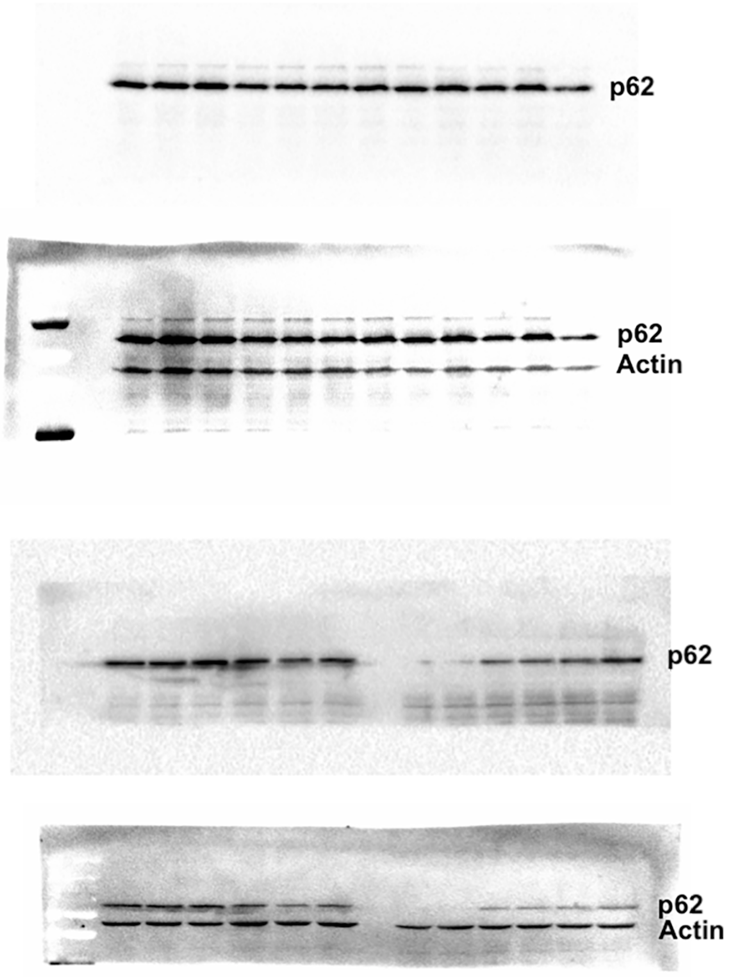

Supplementary Figure 5A – Full blot images

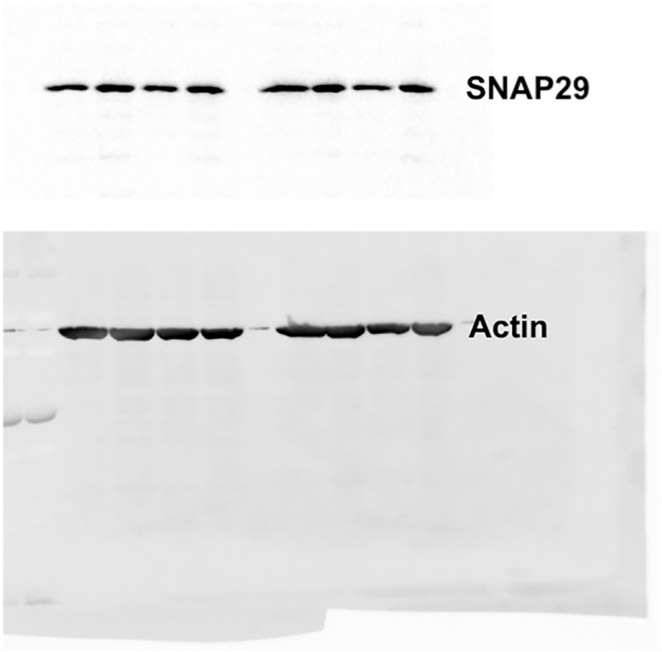

Supplement: S1 File — (PDF) [file pone.0223254.s001.pdf]
