# Supplementary figures and images for: SNARE proteins rescue impaired autophagic flux in Down syndrome
Source: PLoS One. 2019 Nov 12;14(11):e0223254. doi: 10.1371/journal.pone.0223254 (PMC6850524; doi:10.1371/journal.pone.0223254)

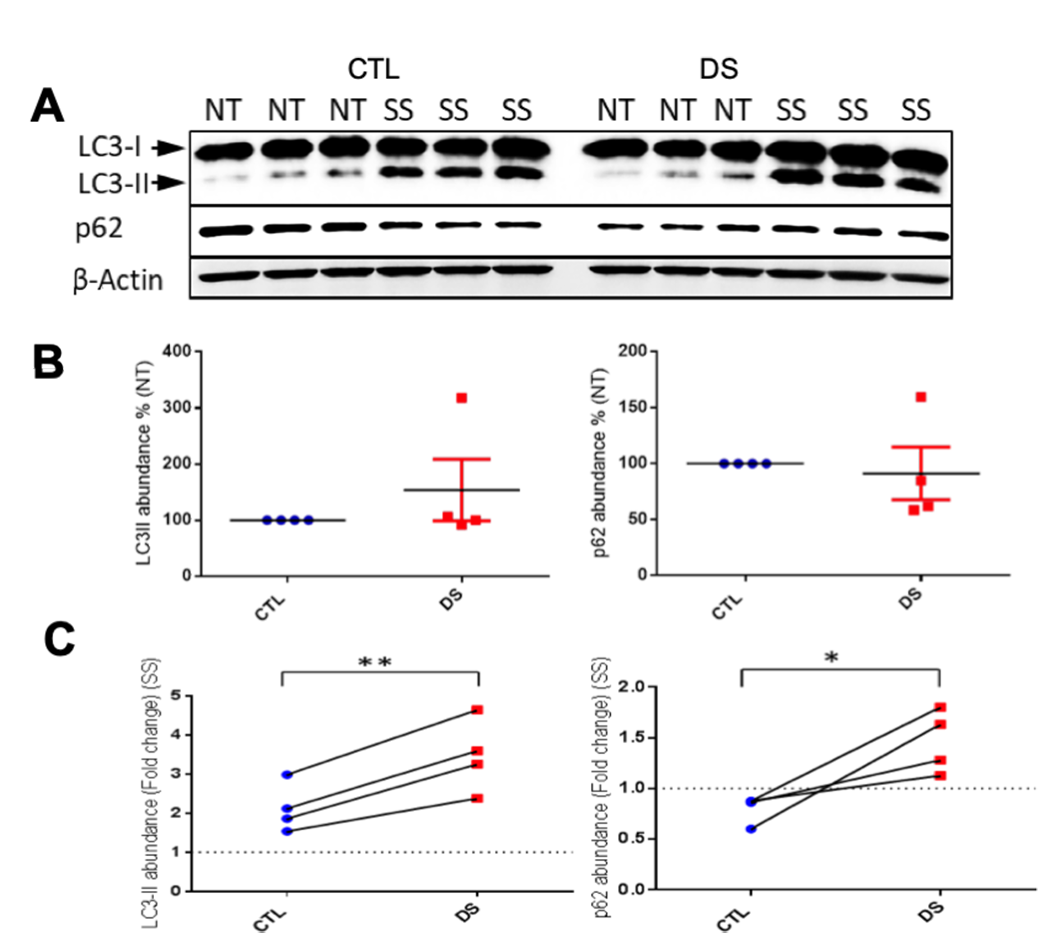

Supplement: S1 Fig — (A) Representative blot of LC3-I, LC3-II, p62 and β-actin of CTL1-4 and DS1-4 fibroblast cell lines (three technical replicates per treatment and genotype of one CTL and DS cell line are presented). (B) Quantification of abundance levels of LC3-II and p62 of four cell line pairs of CTL and DS fibroblasts at basal levels (% based on abundance of CTL NT) (C) Quantification of fold change in abundance of LC3II and p62 of four cell line pairs of CTL and DS fibroblasts after serum starvation (TIF) [file pone.0223254.s002.tif]

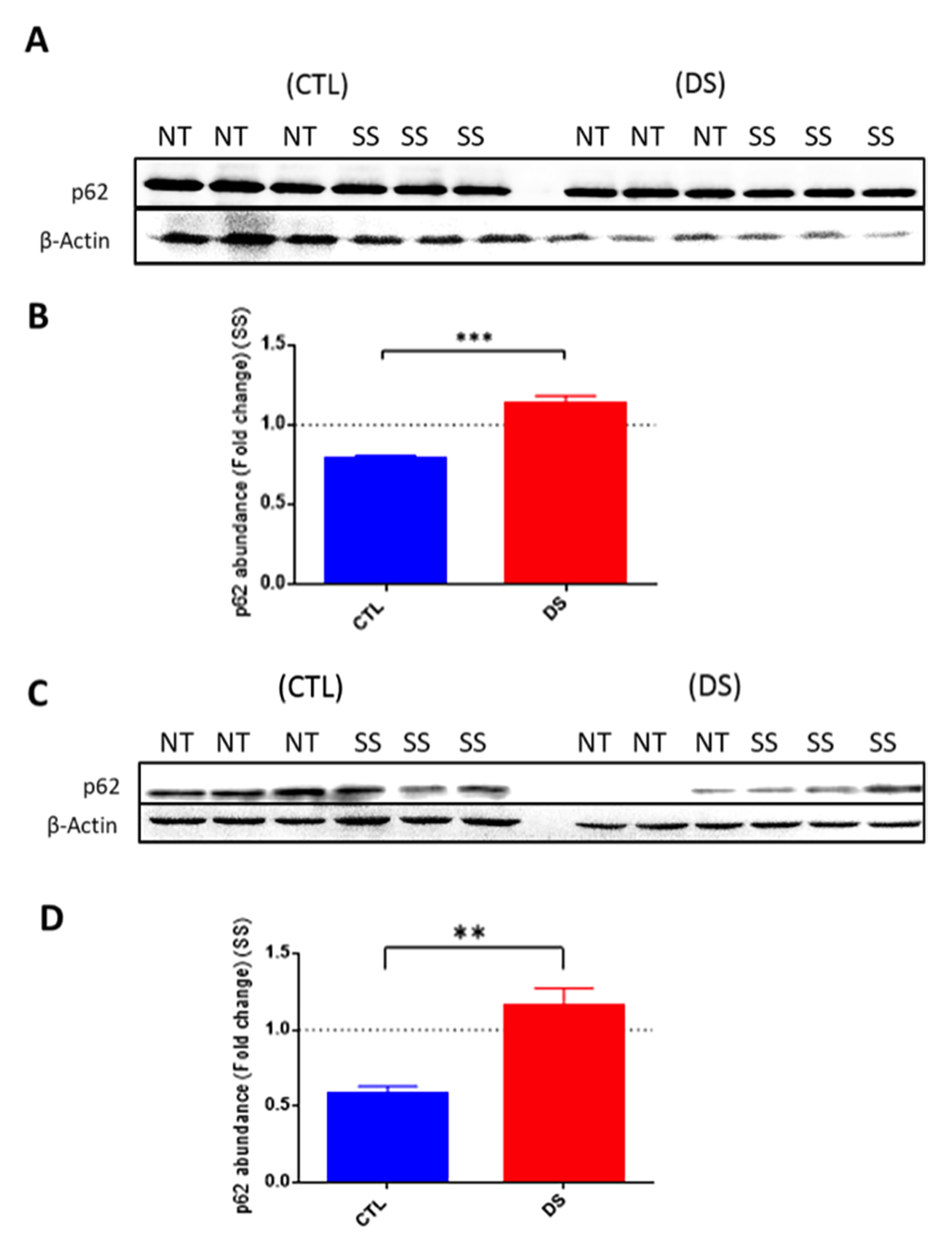

Supplement: S2 Fig — (A) Representative blots for p62 and b-actin of a CTL and DS iPSC cell line at basal levels (NT) or after serum starvation (three technical replicates per treatment and genotype of one CTL and DS cell line are presented). (B) Quantification of fold change in abundance of p62 after serum starvation. (C) Representative blots for p62 and b-actin of a CTL and DS NPC cell line at basal levels or after serum starvation. (D) Quantification of fold change in abundance of p62. (TIF) [file pone.0223254.s003.tif]

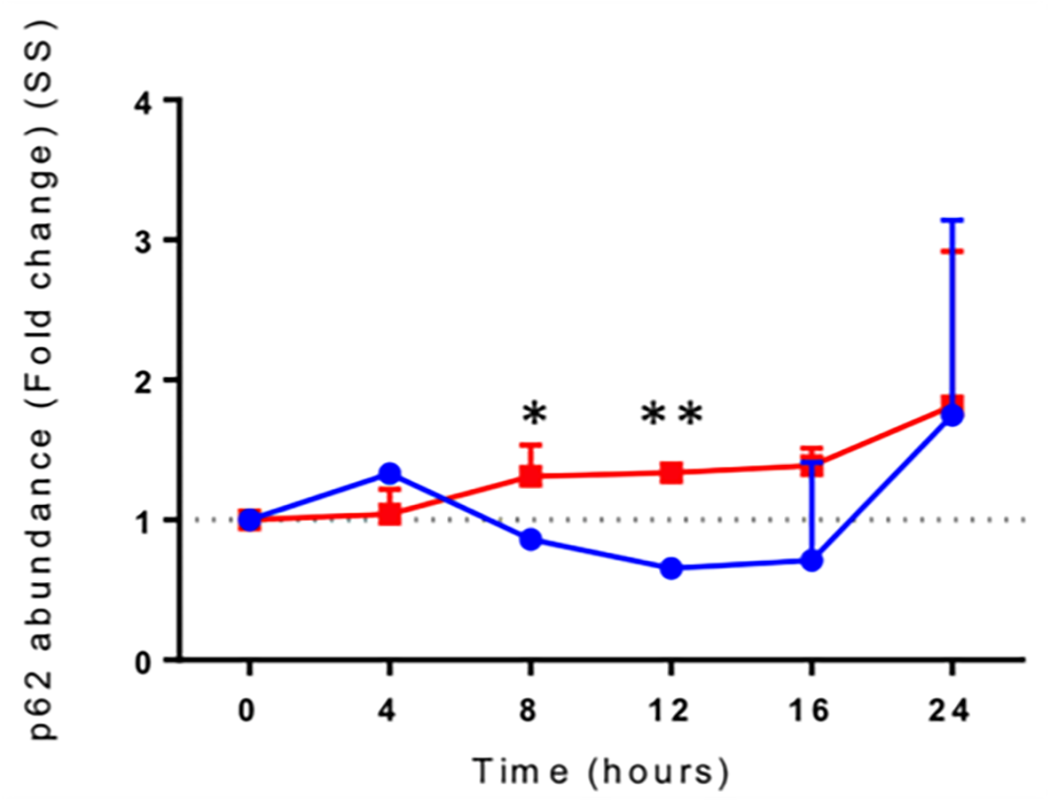

Supplement: S3 Fig — Western blots were conducted over a 24h period to investigate the temporal changes in p62 protein levels after serum starvation in a CTL (AG004392-Blue) and DS (AG006872-Red) fibroblast cell line. Quantification of fold change in abundance of p62 at 0h, 4h, 8h, 12h, 16h or 24h of serum starvation. Statistical analysis was performed by paired t-test analysis at each individual time point. (TIF) [file pone.0223254.s004.tif]

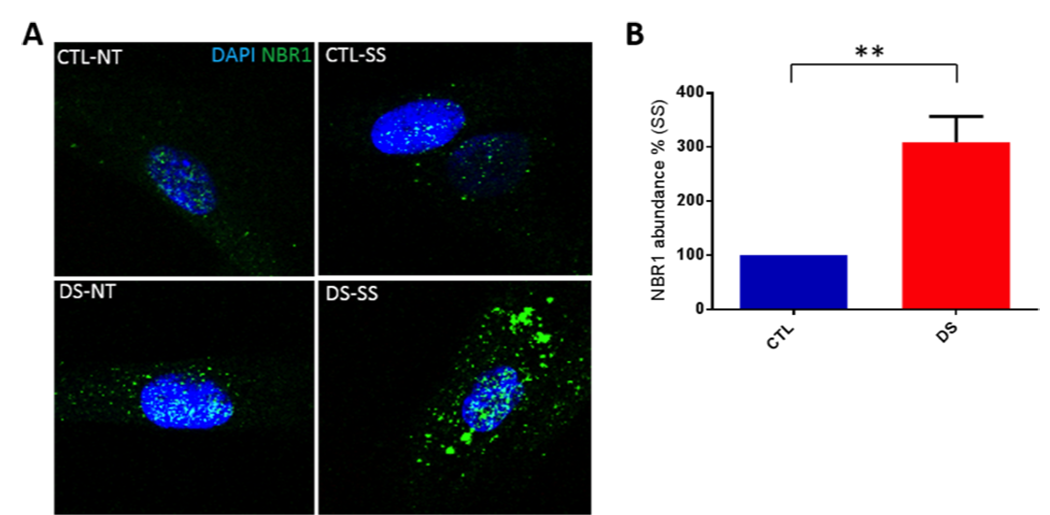

Supplement: S4 Fig — (A) Immunofluorescence for NBR1 (green) and DAPI (blue) in a CTL (AG004392) and DS (AG006872) fibroblast cell line at basal levels or after serum starvation. (B) Quantification of NBR1 (green) fluorescence intensity (area-pixels) after serum starvation (% based on abundance of CTL serum starvation). NT, Not treated-basal levels; SS, Serum starvation (8h). (TIF) [file pone.0223254.s005.tif]

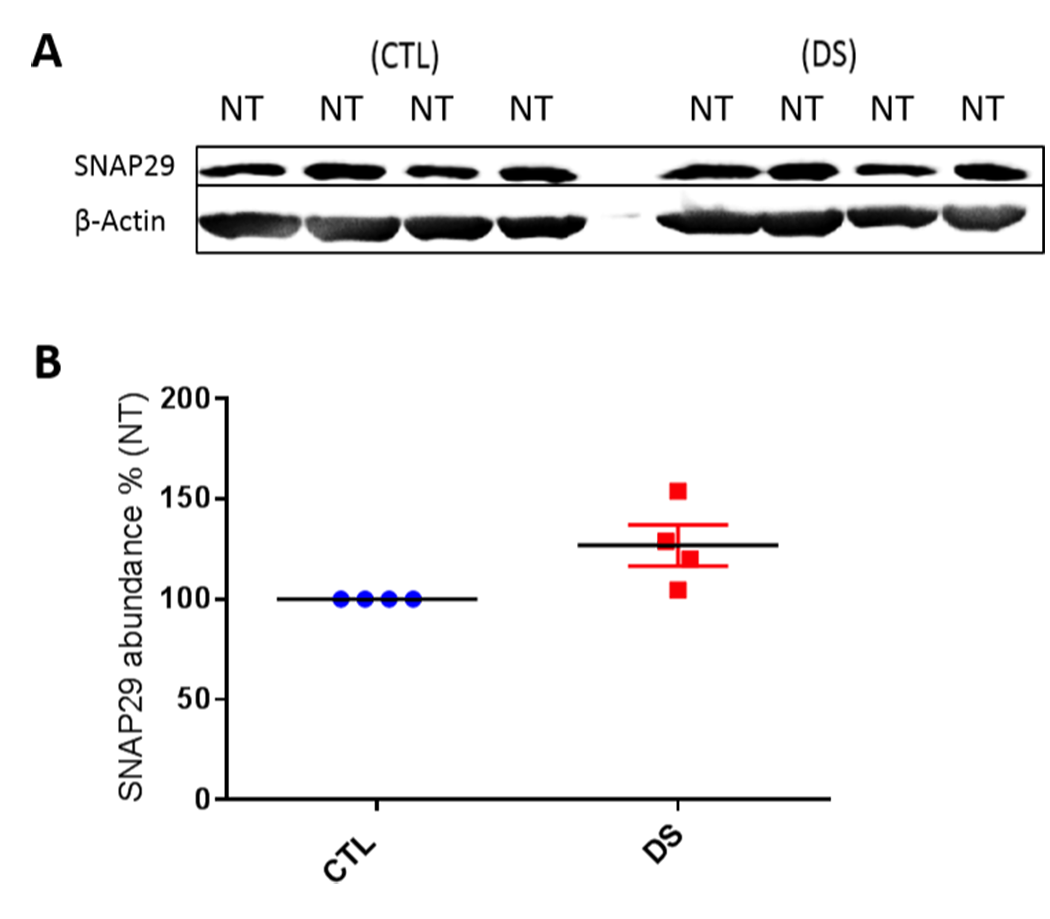

Supplement: S5 Fig — (A) Representative blot for SNAP29 and β-actin in four CTL and DS fibroblast cell lines (CTL1-4, DS1-4) at basal levels. (B) Quantification of SNAP29 levels between CTL and DS groups. NT, Not treated-basal levels (TIF) [file pone.0223254.s006.tif]
